# COVID-19 hotspot detection in a university setting

**DOI:** 10.1371/journal.pone.0289254

**Published:** 2024-05-16

**Authors:** Garrett Duncan, William F. Christensen, Camilla Handley

**Affiliations:** Department of Statistics, Brigham Young University, Provo, Utah, United States of America; University of Massachusetts Lowell, UNITED STATES

## Abstract

The onset of the COVID-19 pandemic commenced an era of widespread disruptions in the academic world, including shut downs, periodic shifts to online learning, and disengagement from students. In an effort to transition back to in-person learning, many universities and schools tried to implement policy that balanced student learning with community health. While academic administrators have little control over some aspects of COVID-19 spread, they often choose to use temporary shutdowns of in-person teaching based on perceived hotspots of COVID-19. Specifically, if administrators have substantial evidence of within-group transmission for a class or other academic unit (a “hotspot”), the activities of that class or division of the university might be temporarily moved online. In this article, we describe an approach used to make these types of decisions. Using demographic information and weekly COVID-19 testing outcomes for university students, we use an XGBoost model that produces an estimated probability of testing positive for each student. We discuss variables engineered from the demographic information that increased model fit. As part of our approach, we simulate semesters under the null hypothesis of no in-class transmission, and compare the distribution of simulated outcomes to the observed group positivity rates to find an initial *p*-value for each group (e.g., section, housing area, or major). Using a simulation-based modification of a standard false discovery rate procedure, we identify possible hot spots—classes or groups whose COVID-19 rates exceed the levels expected for the demographic mix of students in each group of interest. We use simulation experiments and an anonymized example from Fall 2020 to illustrate the performance of our approach. While our example is based on hotspot detection in a university setting, the approach can be used for monitoring the spread of infectious disease within any interconnected organization or population.

## 1 Introduction

Universities have the potential to harbor transmission hotspots (locations or groups with significantly elevated positivity rates) for COVID-19 because of the concentrated population of students and the high number of in-person interactions. Consequently, the COVID-19 pandemic prompted many academic institutions to use alternative modes of instruction to deal with outbreaks. Many college campuses attempted to keep case counts down by shortening semesters, reducing in-person contact time, and periodically moving courses online. Responding to an outbreak with a school closure would likely have negative impacts on community health as thousands of students would disperse into the surrounding communities [1]. While there is a compelling argument that student behavior outside of class is likely more impactful than in-class interactions, if administrators obtained evidence of substantial in-class transmission, commitment to the safety of the campus community might prompt university administrators to move some problematic classes (or all classes) online. Alternatively, administrators might contact individual departments or instructors regarding hotspot sections to mitigate extreme COVID-19 spread rather than moving unnecessarily to campus-wide remote learning. The framework proposed here provides a means for evaluating the risk associated with in-person instruction and identifying and responding to hotspots in statistically rigorous ways.

One challenge associated with detecting hotspots is defining the groups or areas to be measured for elevated positivity rates. Students are enrolled in multiple courses, creating complex interdependencies between the positivity rates across courses. This is magnified when several classes contain a nearly identical group of students (e.g., programs with cohorts). These interdependencies complicate the task of identifying a group as truly a “hotspot.” We thus consider a method based built upon a model for predicting student-level outcomes, using student-level positivity probabilities to evaluate larger groups like sections, housing groups, and majors.

Past approaches for identifying hotspots of infectious disease transmission have utilized tracking devices [2] or social network information [3]. Our approach uses only the standard demographic student information available to the university, along with incoming data on voluntary COVID-19 testing. Using demographic information about university students as predictor variables, we use a statistical model for COVID-19 positivity that produces an estimated probability of testing positive for each student. Using these person-specific probabilities, we can simulate a typical semester (or other time period) for a group (e.g., a class). That is, to evaluate the potential presence of in-class transmission, we simulate group positivity rates under the assumption that new COVID-19 infections within the university community are occurring entirely outside of classroom settings. In other words, for testing sections of a university course or any other groups of interest (e.g., housing locations), we evaluate how the observed group positivity rates differ from those we would expect in a universe with no in-group transmission.

In practice, our analyses often involve performing a large number of simultaneous significance tests (e.g. testing approximately 5,000 courses to identify hotspots). By running multiple significance tests, we run the risk of an inflated false discovery rate (FDR). Benjamini and Hochberg [4] introduced the notion of FDR and the most well-known procedure for controlling the FDR among multiple comparisons. Benjamini and Yekutieli [5] developed a simple, conservative modification of the Benjamini-Hochberg procedure that controls the FDR for dependent test statistics. Our situation is complicated by the dependency between tests (e.g., one student’s behavior affects multiple classes) and the non-uniformity of the distribution of class-level *p*-values. We propose a new simulation-based method to control the FDR that allows for dependent hypothesis tests and requires no assumptions about the distribution of the collection of *p*-values being evaluated.

Due to the large population of students that live in the area surrounding the university we are studying, administration may also have cause to worry about how the spread of the virus within the university might affect the community around it. Hamidi et. al [6] investigated if population density aggravated the pandemic. They used structural equation modeling and ordinary least squares regression to model COVID-19 case counts and death counts. While they mainly focus on population density as an explanatory variable, they use other variables such as age, ethnicity, income, education levels and some health aspects, such as the percentage of smokers in the area. While we do not use any past health data because none were available to us, but there is evidence that these kinds of variables can contribute to vulnerability to COVID-19 and would likely be quite useful [7]. We plan to use demographic characteristics to model the positivity rates (which is a scaled case count) of certain groups. The findings of Hamidi et. al [6] suggest that connectivity (such as economic, social, and commuting relationships) matters more than population density in the spread of COVID-19. We suspect that social connectivity is important in the spread of COVID-19, but do not have the social network data required to incorporate the social networks of individual students, but we use housing information as a partial proxy for social connectivity among students.

**Fig 1:**
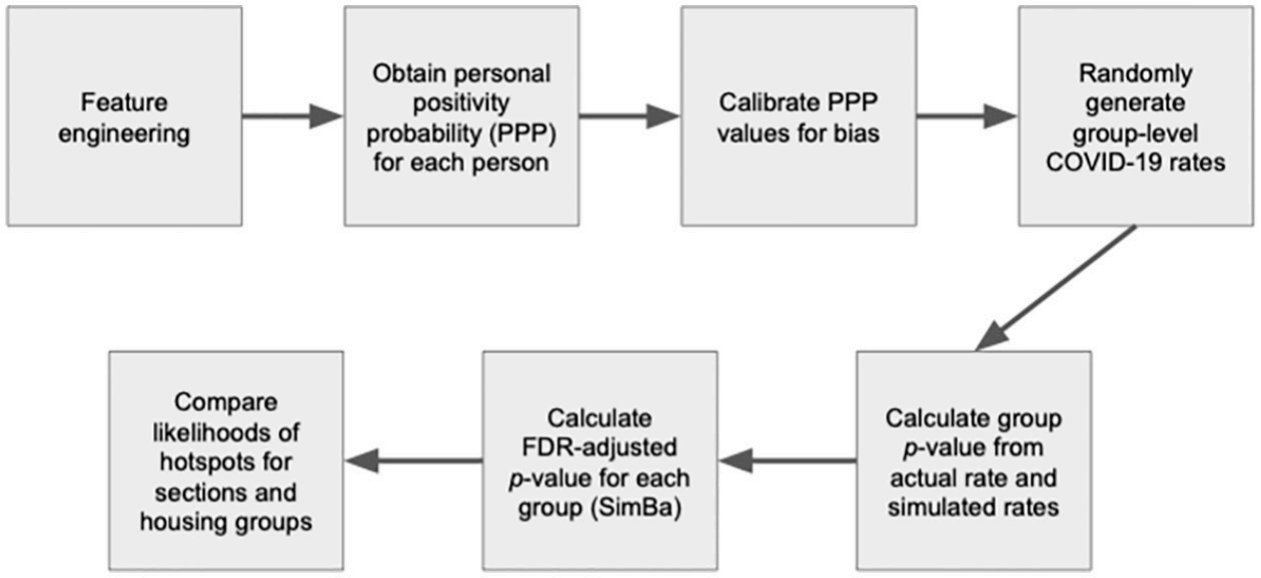
Framework outline. Flow diagram illustrating steps for identifying potential viral-transmission hotspots within potentially-overlapping groups.

Fig 1 illustrates the basic steps in our proposed approach for identifying potential viral-transmission hotspots within potentially-overlapping groups of a population. We begin by building a model for the personal positivity probability (*PPP*_*i*_) of each person. After calibrating the *PPP*_*i*_ estimates, we use the bias-adjusted *PPP*_*i*_ estimates to randomly generate a distribution of positivity rates for each group (under the hypothesis of no in-group transmission). From these distributions of simulated group rates and the actual rate for each group, we obtain a *p*-value for the group, then use a simulation-based FDR adjustment to control the false discovery rate.

The remainder of this article is laid out as follows. Section 2 discusses the engineering of features for use in our statistical modeling and the resulting estimation of personal positivities using extreme gradient boosting (XGBoost). Because our approach involves the simultaneous evaluation of over 5000 sections (or classes), Section 3 introduces and discusses our simulation-based method for false discovery rate (FDR) protection. A simulation experiment illustrating the performance of our FDR method is given in Section 4. In Section 5, we illustrate our methods with an anonymized example from a large university in the western United States. We provide some concluding thoughts in Section 6.

## 2 Modeling of personal COVID-19 positivity

In order to evaluate the health risk associated with membership in any particular group (e.g., a section of a university course or a housing area), we first require an approach for quantifying the personal risk associated with each person ignoring their association with that group. For example, assume that two university sections (classes) have a semester-long positivity rate of 10%. If this were higher than the overall positivity rate for the university, one might naively conclude that each section has elevated rates because of behavioral or structural problems associated with the section (e.g., lax enforcement of mask wearing, poor classroom ventilation, or insufficient physical distancing of class members). However, suppose that we also have the *PPP*_*i*_ for all students (*i* = 1, …, *populationsize*), where *PPP*_*i*_ ranges from 0 (no chance of infection during the current time period) to 1 (certainty of infection). Further, suppose we know that Section 0001 is comprised almost entirely of high-risk students (e.g., single, first-year students who happen to live in housing areas with recently high levels of COVID-19) while Section 0002 is comprised of almost entirely low-risk students (e.g., married, graduate students living in housing areas with recently low levels of COVID-19). In such a scenario, one might flag Section 0002 as a potentially unhealthy environment while declaring Section 0001 as a success for obtaining a positivity rate that is substantially lower than one might have feared, given its high risk collection of students. The key to such is a distinction is the identification of variables that can be used to model a student’s probability of infection.

### 2.1 Feature engineering

As COVID-19 falls in the class of respiratory diseases [7], transmission occurs predominantly from close contact with infected persons. Our entire goal in feature engineering is to use existing data to characterize proximity to other COVID-positive individuals. The rest of this section discusses approaches to this task.

Student demographic and COVID-19 test data have been made available to us by a large university in the western United States. Weekly testing data were obtained from local testing sites, random testing, and self-reports. The data also contain basic demographic and academic information including sex, age, marital status, ethnicity, residential address, hometown zip code, university employment status, number of credit hours completed, major of study, current class schedule, and role at the university (student, faculty, or staff). Due to a non-disclosure agreement between the authors and the university, the data cannot be shared publicly, but inquiries about accessing summary-level data may be directed to the person listed in our data availability statement. The data used contain personal, identifying information of individuals, including address, class schedule, sex, etc. Throughout the analysis, strict standards of privacy were maintained, and the information was only used for their intended purposes.

One of our primary tools for characterizing person-to-person interaction is the residential addresses of students. Christakis and Fowler [3] used social networks to detect the flu, but absent this type of data, we propose using residential proximity to best approximate social connection. Although compliance with public health recommendations about social engagement was lax among some student populations, the practice among some to reduce socializing and conduct work from one’s residence led us to suppose that residential location may be helpful even among those practicing physical distancing.

After cleaning the data using R software, we converted residential addresses from character data to latitude/longitude coordinates. This geocoding was done with the Bing API [8]. Using these coordinates, we created an *N* × *N* inter-student distance matrix *H* for *N* total students, with elements defined as *h*_*ij*_ = ||*S*_*i*_ − *S*_*j*_||, the distance in miles between two students *S*_*i*_ and *S*_*j*_. This distance matrix is the backbone of several important features we created. To more readily capture important facets of the data provided by the University, we constructed new features from the initial set of variables, including:

- *Minimum Distance (MD_it_)*: the distance between student *i* and the closest student testing positive during week *t*, or
  $$\underset{i}{\text{min}}\left\{H_{ij}:S_{j}\;\text{tested}\;\text{positive}\;\text{during}\;\text{period}\;\text{of}\;\text{interest}\right\}$$
- *Percent within d feet (Pd_it_)*: for a chosen distance, *d*, the percent of students residing within *d* feet of student *i* testing positive during week *t*. Sensitivity analyses led us to the use of *P*20_*it*_, or the percent within 20 feet (6.1 meters). Note that *Pd*_*it*_ is similar to *MD*_*it*_, but captures local density of infection rather than proximity to infection. A weakness of this metric is that, depending on how mailing addresses are assigned (e.g., in large apartment complexes), some students may have hundreds of people “within 20 feet.”
- *Housing Positivity (HP_it_)*: percent of student *i*’s housing group testing positive during week *t*, where a housing group is a group of typically 2000-8000 students sorted geographically using major dormatory groups or geographic regions within the university’s city.
- *Major Positivity (MP_it_)*: percent of student *i*’s academic major group testing positive during week *t*.
- *Dead Time (DTb_i_)*: a measure of how long student *i* might spend on campus, but not in class; *DTb*_*i*_ is the sum across Monday through Friday of all same-day time gaps (*b* hours or less) between two of student *i*’s classes. This measure assumes that a student will stay on campus somewhere (and potentially in social environments) if they have another class that follows in *b* hours or fewer. This feature attempts to capture potential student interaction on campus, but outside of the classroom. Using sensitivity analyses we use *DT*2_*i*_. For example, a student with MWF classes at 9 am and Noon, and TTh classes at 9 am, 2 pm, and 3 pm, *DT*2_*i*_ = 6 (2 hours of dead time on MWF and 0 hours of dead time on TTh).
- *Home County Politics*: the difference in Biden and Trump voting percentages of a student’s home county in the 2020 presidential election.

Some variables were considered to be fixed throughout the semester (e.g., marital status and sex) while others changed in value from week to week depending on incoming reports of COVID positivity (e.g., *P*20_*it*_, *MD*_*it*_, *HP*_*it*_, and *MP*_*it*_). While it may seem natural to use the most recent week’s values to predict the upcoming personal positivity probability *PPP*_*i*_ for the next week, we considered how additional summaries of the weekly values for these features could be used in the model. For example, given that we are 6 weeks into the semester, should we use last week’s value of “Minimum Distance” for each student or the worst week in the semester for “Minimum Distance”, or both? Across the features that we calculated on a per week basis, we created three ways to organize and test the performance of these features:

- The most recent week, denoted with suffix *r*. For example, if currently in week *t*, *MPr*_*i*_ = *MP*_*it*−1_ for student *i*.
- The worst or extreme week, denoted with the suffix *w*. For example, *HPw*_*i*_ is the worst measured housing positivity in earlier weeks of the semester for student *i*, or max{*HP*_*i*1_, *HP*_*i*2_, …, *HP*_*it*_} for week *t*.
- The median value week, denoted with suffix *m*. For example, *MDm*_*i*_ is the median minimum distance in earlier weeks of the semester for student *i*, or median{*MD*_*i*1_, *MD*_*i*2_, …, *MD*_*it*_} for week *t*.

Sensitivity analyses indicate that for the features *P*20_*it*_, *MD*_*it*_, *HP*_*it*_, and *MP*_*it*_, the three summaries of the history of these values (worst, median, and recent) are each helpful and often provide additional predictive power when used in the presence of the others. Evaluating the effectiveness of these features will be discussed in Section 2.2.

### 2.2 Statistical modeling of personal positivity probabilities

Our next step is to use a statistical model to estimate a student’s personal positivity probability (*PPP*_*i*_) given training data from the time window of interest (e.g., all past weeks in the current semester or the past 6 weeks). Instead of initially focusing on course level metrics or statistics, we emphasize the risk each student carries (with the *PPP*_*i*_), creating more flexibility in evaluating groups. This allows the collective risk of a group to be formed from its individual members, which is especially helpful when considering how students are enrolled in multiple courses.

Each week we compile a data set to be used in training the model. Let *Y*_*i*_ be the test status (response) variable defined as the COVID status for that student (*Y*_*i*_ = 1 indicates that person *i* tested positive during the time window of interest, and *Y*_*i*_ = 0 indicates otherwise). The predictor variables are listed in the first column of Table 1. Detailed descriptions of the engineered variables can be found in Section 2.1. Note that because we wish to evaluate the potential for specific classes to be hotspots, this list of variables excludes any information about enrollment in any section. Similarly, if we wished to examine the potential for hotspots in housing areas, class standing groups (Fr/So/Jr/Sr/Grad), or any other groups, our models for *PPP*_*i*_ would not include variables variables associated with those groups.

**Table 1:** Variable variable summary. Explanatory variable names and average gain for variables used by XGBoost to predict COVID positivity during the time window of interest. The given variable importance scores correspond to the XGBoost fit for data in Week 15 when the full semester is the time window of interest. Engineered features are listed in black, and basic information given from the university are shown in blue.

Feature	Gain
*DT*2 (sum across dead time gaps of ≤ 2 hours)	0.1019
Home County Politics (difference in Biden minus Trump)	0.1008
*P*20*w* (% within 20 feet during worst week)	0.0951
*MDm* (median minimum distance to positive of past weeks)	0.0906
*MDr* (minimum distance to positive during recent week)	0.0803
*HPw* (worst recorded housing positivity in past weeks)	0.0695
*HPm* (median housing positivity of past weeks)	0.0614
*MPr*	0.0547
Age	0.0544
*MDw*	0.0412
*P*20*m*	0.0251
*P*20*r*	0.0150
*MPm*	0.0145
*MPw*	0.0141
Employed by University	0.0137
Sex A	0.0118
Class Standing A	0.0105
Marital Status C	0.0098
Class Standing D	0.0090
*HPr*	0.0089
Marital Status A	0.0087
Class Standing C	0.0085
Class Standing E	0.0081
Ethnicity F	0.0076
Ethnicity D	0.0075
Housing Group J	0.0060
Marital Status B	0.0058
Zip Code Missing (indicator)	0.0057
Housing Group L	0.0054
Housing Group C	0.0051
Housing Group G	0.0051
Sex B	0.0049
Housing Group F	0.0042
Ethnicity H	0.0033
Ethnicity I	0.0032
Athlete	0.0032
Class Standing F	0.0032
Housing Group B	0.0030
Housing Group A	0.0025
Class Standing B	0.0021
Ethnicity E	0.0021

Variables with a Gain less than 0.0021 are not displayed such as Housing Group D, Housing Group H, Housing Group K, Ethnicity G, Housing Group E, Ethnicity A, Ethnicity C, Sex C, Ethnicity B, and Housing Group I.

We then use Extreme Gradient Boosting (XGBoost) in R [9, 10] to predict the COVID status for each student. XGBoost is a boosted decision tree algorithm that facilitates the evaluation of feature importance and generally predicts more accurately than logistic regression and other competing methods for these data. Through *K* = 6 fold cross validation, we tested several combinations of hyperparameters to determine the model with the highest out-of-sample AUC.

From the XGBoost model, we obtain the probability of COVID positivity for each person (*PPP*_*i*_). When the time period is the whole semester, the probabilities achieved an in-sample and out-of-sample AUC of 0.9634 and 0.6959, respectively. Table 1 provides variable importance scores for each of the variables in the model. Values in the gain column are the average feature contribution to the model during training in the last week of the semester across the 6 folds. Though the calculation for measuring gain can be complicated, it summarizes how much entropy is decreased in predicting COVID-19 status when a split is made using that variable weighted by how many observations were affected by the split. The five most valuable predictors of a person’s probability for contracting COVID-19 included an approximation of the amount of “dead time” spent on campus between classes, Home County Politics, the worst measure of *P*20, and two historical measures of the distance to the nearest positive *MD*.

Because many of the predictor variables are one-hot-encoded levels of a categorical variable (e.g., “Sophomore” and “Senior” as Bernoulli variables indicating levels for class standing), we consider in Table 2 the sum of the variable importances for all one-hot-encoded variables associated with a particular categorical variable. For example, 0.0414 represents the total gain summed across all of the variables associated with class standing. Note that even after summing the gains associated with the levels of each categorical variable, only class standing is comparable in value to the top 10 feature importances.

**Table 2:** Categorical variable importance totals. Sum of variable importances for all levels of a given categorical type.

Feature	Total Gain
Class standing	0.0414
Housing Group	0.0382
Ethnicity	0.0285
Marital Status	0.0243
Sex	0.0176

**Fig 2:**
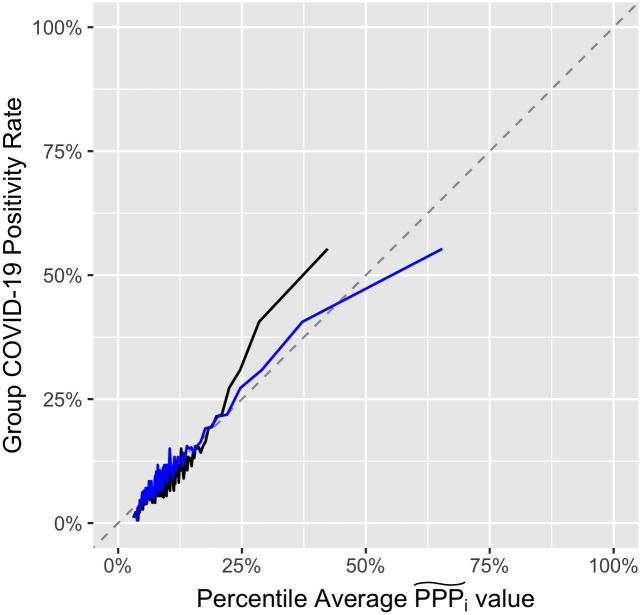
COVID-19 rate and personal positivity probability percentiles. Actual COVID-19 rates within each of the 100 percentile-group of the population, ordering by the XGBoost-derived personal positivity probabilities (${\tilde{PPP}}_{i}$) in black. The bias-adjusted probabilities (${\hat{PPP}}_{i}$) are shown in blue.

Fig 2 illustrates the relationship between the XGBoost probabilities (${\tilde{PPP}}_{i}$) and the actual rate of COVID-19 among the 100 percentile-groups of the student population, ordered by ${\tilde{PPP}}_{i}$. Note that while the actual COVID-19 rate for each percentile group tends to be positively correlated with the average ${\tilde{PPP}}_{i}$ value within each group, the rates are not monotonically increasing with average ${\tilde{PPP}}_{i}$ and the ${\tilde{PPP}}_{i}$ values exhibit a small negative bias for the actual rates. Thus, we calibrate the ${\tilde{PPP}}_{i}$ values via a probit regression of COVID status on ${\tilde{PPP}}_{i}$, as shown in Eq 1.

$$\begin{array}{c} {PPP}_{i}=P\left(Y_{i}=1\right)=\Phi \left(\beta_{0}+\beta_{1}{\tilde{PPP}}_{i}\right) \end{array}$$ (1)

where *Y*_*i*_ indicates the positivity status for person *i* (1 for COVID-19 positive, 0 otherwise), Φ is the cumulative distribution function of a standard normal, and *β*_0_ and *β*_1_ are parameters solved for using maximum likelihood estimation. These bias-adjusted predicted probabilities arising from the probit regression (${\hat{PPP}}_{i}$) are then used as our person-specific estimates of *PPP*_*i*_.

## 3 Quantifying evidence of hotspot potential

### 3.1 Simulation-based method for obtaining a *p*-value for each group

In order to quantify the evidence for a group being a hotspot, we use a *p*-value calculated from the observed positivity rate for that group. Specifically, our *p*-value is the estimated probability of observing a group positivity at least as high as the one actually observed, if in fact there is no in-group transmission. To obtain this *p*-value, we simulate semesters under the null hypothesis of no within-group transmission, and compare the distribution of simulated outcomes to the observed group positivity rates to find an initial *p*-value for each group (e.g., section, course, or major). That is, for each of 1000 simulated semesters (or other time period), we generate 1,000 time periods (e.g., semesters) of student COVID status outcomes using the ${\hat{PPP}}_{i}$ value for each student. Specifically, for each simulated time period, the *i*th student’s COVID-19 status is randomly generated with

$$\begin{array}{c} y_{i}∼\text{Bern}\left({\hat{PPP}}_{i}\right), \end{array}$$ (2)

so that in the simulated time period, the *i*th student’s status (*y*_*i*_) is either a 1 (COVID positive) or a 0 (COVID negative), *i* = 1, …, *N*, and *N* is the number of students in the population. These individual student status values provide a means for evaluating expected positivity rates for any group.

**Fig 3:**
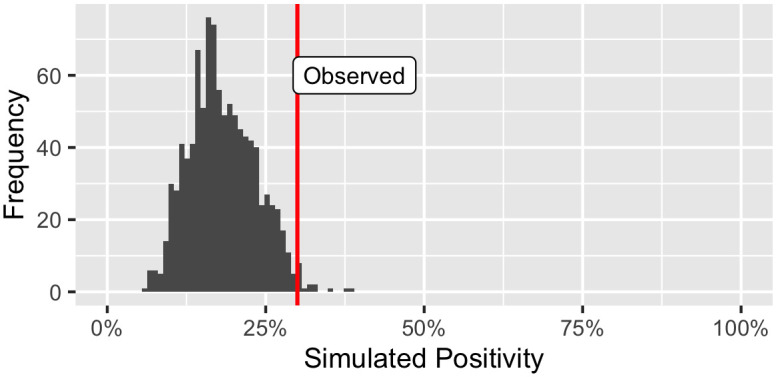
Example null distribution of simulated rates. Histogram of simulated rates for an example class under the null hypothesis of no in-class transmission. The red, vertical line indicates the actual observed rate of Fall 2020 for this section.

To determine if there is a significant hotspot among a group of students (e.g., a section of a university course or a housing group), we obtain a *p*-value derived from the simulated semesters from the simulated student status’ belonging to that group. Let a group of interest be denoted *G*_*j*_, *j* = 1, …, *N*_*G*_, where *G*_*j*_ may be a group of students enrolled in a section of a university course or declared as a specific major, and *N*_*G*_ is the total number of sections or majors being considered. In this article, we consider only an evaluation of class sections and housing groups. For the *j*th group of students, we find the *p*-value:

$$\begin{array}{c} p_{j}=Pr\left({POS}_{sim,j}\geq {pos}_{obs,j}\right), \end{array}$$ (3)

where *POS*_*sim*,*j*_ represents the positivity of a possible simulated semester for group *j* and *pos*_*obs*,*j*_ is the observed positivity rate for that group. For a given group *G*_*j*_, the empirical distribution of *POS*_*sim*,*j*_ represents the null distribution, or the distribution of COVID-19 positivity rates we could reasonably expect under the assumption there is no in-group spread already occurring. An example of the null distribution for a group is shown in Fig 3. The red, vertical line indicates the actual observed rate for that group. The *p*-value for this group would be low, as there are few simulated semesters greater than the actual rate of COVID-19 in this section. A *p*-value close to 0 indicates high severity, or a high likelihood of in-group transmission in that group. When testing university course sections—our primary interest here—this *p*-value tests

$$H_{0_{j}}:\text{There}\;\text{is}\;\text{no}\;\text{in-class}\;\text{transmission}\;\text{happening}\;\text{in}\;\text{class}\;j.$$

but similar null hypotheses can be applied to other groups of interest (e.g., majors, housing groups, etc.). As this is done for groups *G*_*j*_, *j* = 1, …, *N*_*G*_, we raise the probability of an inflated group-wise Type I error rate through multiple testing. To account for this, we employ a *p*-value correction method to control the False Discovery Rate (FDR).

### 3.2 Simulation-based method for controlling false discovery rate

Benjamini and Hochberg [4] introduced one of the most frequently-used procedures for controlling the False Discovery Rate (FDR) in a setting involving numerous tests. Consider testing $H_{0_{1}},H_{0_{2}},\ldots ,H_{0_{N_{G}}}$ based on the corresponding *p*-values $p_{1},p_{2},\ldots ,p_{N_{G}}$. Let $p_{\left(1\right)}⩽p_{\left(2\right)}⩽\ldots ⩽p_{\left(N_{G}\right)}$ be the ordered *p*-values, and denote by *H*_(*j*)_ the null hypothesis corresponding to *p*_(*j*)_. Assume that of the *N*_*G*_ hypotheses tested, $N_{G_{0}}$ of them are true null hypotheses. Define the following multiple-testing procedure for a scenario in which the desired FDR is set to *q* (0 < *q* < 1):

$$\begin{array}{c} \begin{array}{c} \text{Let}\;k\;\text{be}\;\text{the}\;\text{largest}\;j\;\text{for}\;\text{which}\;p_{\left(j\right)}⩽\frac{j}{N_{G}}q;\\ \text{then}\;\text{reject}\;\text{all}\;H_{\left(j\right)}\;\text{for}\;j=1,2,\ldots ,k. \end{array} \end{array}$$ (4)

For independent test statistics and for any configuration of false null hypotheses, the above procedure controls the FDR at $q\cdot \frac{N_{G_{0}}}{N_{G}}⩽q$ [4]. We will refer to this method as the BH method.

In addition to the independence requirement, this method is based upon the assumption that the *p*-values are uniformly distributed under the null hypotheses [5]. We expect that the *p*-values that we will collect from each group (i.e. section or course) will be dependent due to the fact that each student is generally enrolled in more than one course, so a single student’s behavior affects the positivity rate and potential transmission risk of all the courses they are enrolled in. Furthermore, as discussed later, examination of the *p*-values associated with this non-standard testing scenario indicates clear non-uniformity. Benjamini and Yekutieli [5] develop a method that controls the FDR for dependent test statistics. We will refer to this method as the BY method. They proved that when the BH procedure is conducted with *q*/($\sum_{j=1}^{N_{G}}\frac{1}{j}$) taking the place of q in Eq 4, the FDR can still be maintained at a level less than or equal to $q\cdot \frac{N_{G_{0}}}{N_{G}}$. Due to the fast-growing nature of the harmonic number ($\sum_{j=1}^{N_{G}}\frac{1}{j}$), the significance threshold quickly becomes very small as *j* increases, resulting in an extremely low number of significant tests. Benjamini and Yekutieli [11] use resampling of the data to estimate the distribution of the *p*-values. Genovese et. al [12] present a method for hypothesis testing that controls the FDR while incorporating prior information about each hypothesis. They use this prior information by weighting the *p*-values. This method assumes independence of *p*-values, although the asymptotic behaviors pave the way for more general results for dependent or not identically distributed *p*-values. Our intent here is to propose a new method—associated with our simulation-based approach for obtaining group-level *p*-values—that is less conservative than the BY method, with no distributional assumptions.

**Fig 4:**
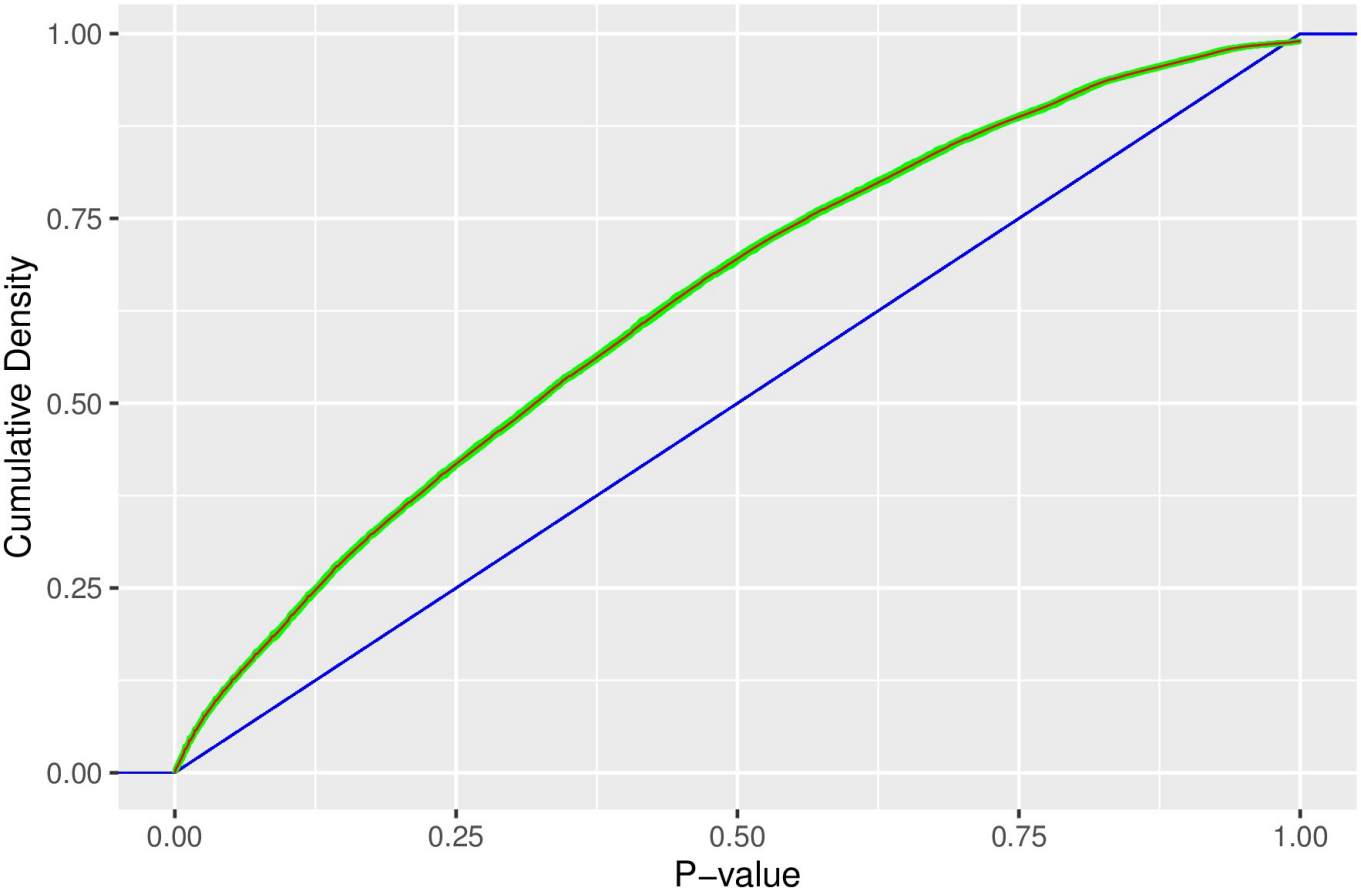
Empirical *p*-value distribution. The estimated *p*-value distribution from 1,000 simulated semesters (green lines and mean in red) along with the uniform distribution function (blue line).

Not only will our *p*-values be dependent, but we also observe that they do not meet the uniformity requirement under the null hypothesis. When testing many hypotheses simultaneously, the breakdown of this uniformity condition (*p*_*j*_ ∼ Unif(0,1) for *j* = 1, …, *N*_*G*_) may cause multiple testing procedures to perform poorly [13]. To account for the violation of the independence and uniformity assumptions in the distribution of *p*-values, we propose a new method that controls the false discovery rate using a simulation-based estimate of the *p*-values’ null distribution. The BH method is defined in (4) by scaling the desired FDR rate (q) by $\frac{j}{N_{G}}$, which is the $\frac{j}{N_{G}}th$ quantile of the distribution of the *p*-values when uniformly distributed. To properly characterize the actual distribution of the *p*-values under the null hypothesis, we simulate 1,000 semesters under the null hypothesis (i.e., under the assumption that there is no in-group transmission), calculating the *p*-values for each group (e.g., class section) in each simulated semester using the methods described in Section 3.1. For the *r*th simulated semester, we obtain the ordered *p*-values ($p_{(1)r}^{*},p_{(2)r}^{*},\ldots ,p_{(N_{G})r}^{*})$. We then estimate the null distribution of ordered *p*-values with (${\bar{p}}_{\left(1\right)}^{*},{\bar{p}}_{\left(2\right)}^{*},\ldots ,{\bar{p}}_{\left(N_{G}\right)}^{*}$), where ${\bar{p}}_{\left(j\right)}^{*}=1/1000\sum_{r=1}^{1000}p_{(j)r}^{*}$. Fig 4 illustrates the estimated cumulative distribution function (CDF) of the distribution of *p*-values, where the green lines represent the CDFs for the *p*-values in each of the 1,000 simulated semesters and the red line indicates the average across all simulations (${\bar{p}}_{\left(1\right)}^{*},{\bar{p}}_{\left(2\right)}^{*},\ldots ,{\bar{p}}_{\left(N_{G}\right)}^{*}$). The CDF for the uniform distribution function is shown in blue. We note the estimated null distribution of *p*-values (red line) deviates markedly from the uniform distribution with a higher concentration of small values (e.g. < 0.25).

We define our simulation-based FDR-controlling method in Eq 5. Consider testing $H_{1},H_{2},\ldots ,H_{N_{G}}$ based on the corresponding *p*-values $p_{1},p_{2},\ldots ,p_{N_{G}}$. Let $p_{\left(1\right)}⩽p_{\left(2\right)}⩽\ldots ⩽p_{\left(N_{G}\right)}$ be the ordered *p*-values, and denote by *H*_(*j*)_ the null hypothesis corresponding to *p*_(*j*)_. Define the following multiple-testing procedure:

$$\begin{array}{c} \begin{array}{c} \text{Let}\;k\;\text{be}\;\text{the}\;\text{largest}\;j\;\text{for}\;\text{which}\;p_{\left(j\right)}⩽{\bar{p}}_{\left(j\right)}^{*}q;\\ \text{then}\;\text{reject}\;\text{all}\;H_{\left(j\right)}\;\text{for}\;j=1,2,\ldots ,k \end{array} \end{array}$$ (5)

where ${\bar{p}}_{\left(j\right)}^{*}$ estimates the $\frac{j}{N_{G}}th$ quantile of the estimated null distribution of the *p*-values. We expect this procedure to control the FDR at *q* for non-uniform and dependent *p*-values. We will refer to this simulation-based method as the SimBa method and consider its performance in the next section.

## 4 Simulation

To test the validity of our simulation-based FDR controlling method, we simulated in-class transmission and compared the accuracy of the BH method, the BY method and our simulation-based method in terms of identifying problematic classes. We performed these simulations using the same data structure as the real data provided by the university, with university courses as the chosen group.

In each simulation we randomly chose a number of courses (among those with at least 10 students) to simulate in-group transmission by increasing the enrolled students’ probabilities of testing positive. We will refer to this as spiking the courses. We varied the number of courses we spiked to see the behavior of the methods as transmission increases. For each course that was spiked, we multiplied the probability of testing positive for each student in the course by a spike factor (*SF*), and capped these probabilities at 1. We considered multiple values for the spike factor to see how much of an increase in probability is necessary for the methods to identify the transmission. We then created an indicator for each student that denotes whether or not the student’s probability had been spiked. We did this to account for and potentially identify the additional spread that would inevitably happen outside of the spiked courses. For each course—regardless of whether or not the course was randomly selected to be spiked, we calculated the percentage of students that were spiked in each course. We then simulate a semester for person *i* by drawing

$$\begin{array}{c} y_{i}∼\text{Bern}\left(\text{min}\left\{s_{i}{\hat{PPP}}_{i},1\right\}\right), \end{array}$$ (6)

where Bern(*π*) denotes the Bernoulli distribution with probability of success equal to *π*, ${\hat{PPP}}_{i}$ represents the estimated probability of testing positive that our model produced for the *i*^*th*^ student, and

$$\begin{array}{c} s_{i}=\{\begin{array}{cc} SF & i^{th}\;\text{student}\;\text{is}\;\text{enrolled}\;\text{in}\;\text{a}\;\text{spiked}\;\text{course}\\ 1 & \;\text{otherwise.} \end{array} \end{array}$$ (7)

Thus, depending on whether or not the *i*^*th*^ student is enrolled in a course that was chosen to be spiked, *s*_*i*_ either has no effect on the probability or it increases it by a predetermined factor (*SF*). To select the spike factors to be used in the simulations, we compared the COVID-19 rate of flagged hotspots to the campus-wide COVID-19 rate at the time that each hotspot was flagged. Most spike factors ranged from 2 to 12, with values on the higher end usually associated with small (more erratic) sections. In our simulations, we use spike factors of 2 or 4 to represent modest to serious hotspots relative to Fall 2020 data for class sections.

After obtaining the simulated positivity rates for each course, we calculated each course’s *p*-value using the processes described in the previous section and compared the three methods in terms of false discovery rate (FDR) and sensitivity. In this case, sensitivity represents the proportion of problematic courses that were identified (or “flagged”) by the given method as problematic. The FDR represents the proportion of courses that were identified as problematic that were in fact not problematic. We calculated the FDR and sensitivity under two definitions of a “true positive” or problematic course. The first definition is the most straightforward: courses that were randomly selected to be spiked. We acknowledge from the outset that none of the methods is expected to strictly protect against false discovery inflation (as it relates to the set of spiked courses) because of the nature of the spiking process and the strong dependency among some classes. For example, if most of the students currently in Math 101 are also in CompSci 101, then a hotpot emerging in Math 101 (i.e., spiking the class in a simulation) will likely lead to a “false discovery” of a problematic environment in CompSci 101. In practice, these dependencies are often apparent (e.g., observing a set of courses associated with a single cohort of students), and the candidate classes for the true source of the disease spread might be decipherable by comparing positivity rates and common student membership within the possible cluster of classes. No such automated method for within-cluster examination is used in this simulation. Notwithstanding, our simulation still allows us to compare the FDR and sensitivity for competing methods when addressing the multiple testing problem.

Because of these complexities associated with the hotspot detection problem (and our simulation) in a university setting, we also consider a second definition of a “problematic course”: a course in which at least 33% of the students enrolled had spiked probabilities. This second definition results in more courses labeled as true positives and should result in lower values for both FDR and sensitivity.

Table 3 shows the different scenarios under which we performed our simulations. We performed 500 replications (i.e., simulated semesters) for each scenario and averaged the results across replications. Each scenario was performed with the desired FDR fixed at 30%. The courses spiked indicates the number of courses that we randomly chose to imitate in-course transmission. These courses were randomly selected from the Fall 2020 semester course list, excluding those that had less than 10 students.

**Table 3:** Simulation design. Simulation scenarios studied using three levels of the number of courses spiked and two levels of the spike factor.

Scenario	Courses Spiked	Spike Factor (*SF*)
1	10	4
2	30	4
3	75	4
4	10	2
5	30	2
6	75	2

### 4.1 Simulation results

Tables 4 and 5 show the results from these simulations and compare the performance of the three methods. The column labeled “# Flagged” indicates how many courses the given method identified as problematic (having significantly high in-class transmission) averaged across all simulations under the given scenario. The “FDR” and “Sensitivity” columns show the false discovery rate and sensitivity for the given method where the true problematic courses are those selected for spiking (i.e., where all the students enrolled have spiked probabilities). Similarly, the “FDR (33%)” and “Sensitivity (33%)” columns show the respective metrics where the true problematic groups are defined as those where at least 33% of the students enrolled have spiked probabilities.

**Table 4:** FDR simulation results with a spike factor of 4. Number of problematic courses found, false discovery rate and sensitivity for competing FDR methods when the spike factor equals 4. “FDR (33%)” and “Sensitivity (33%)” describe the performance of FDR methods among courses with at least 33% of students spiked. Numbers that are italicized indicate a significant difference (0.001 level) from the SimBa method using a t-test.

Courses Spiked	Method	# Flagged	FDR	Sensitivity	FDR (33%)	Sensitivity (33%)
10	BH	29.14	*0.54*	*0.71*	*0.39*	*0.45*
	BY	*16.81*	0.40	0.62	0.23	0.38
	SimBa	17.44	0.40	0.62	0.23	0.38
30	BH	*138.00*	*0.69*	*0.80*	*0.53*	*0.53*
	BY	*57.00*	*0.45*	*0.63*	*0.25*	*0.38*
	SimBa	71.22	0.51	0.68	0.32	0.42
75	BH	*553.647*	*0.81*	*0.88*	*0.65*	*0.62*
	BY	*212.70*	*0.61*	*0.70*	*0.40*	*0.40*
	SimBa	330.00	0.71	0.80	0.51	0.50

**Table 5:** FDR simulation results with a spike factor of 2. Number of problematic courses found, false discovery rate and sensitivity for competing FDR methods when the spike factor equals 2. “FDR (33%)” and “Sensitivity (33%)” describe the performance of FDR methods among courses with at least 33% of students spiked. Numbers that are italicized indicate a significant difference (0.001 level) from the SimBa method using a t-test.

Courses Spiked	Method	# Flagged	FDR	Sensitivity	FDR (33%)	Sensitivity (33%)
10	BH	*5.55*	0.52	0.17	0.50	0.10
	BY	*4.4*	0.50	0.15	0.42	0.09
	SimBa	4.4	0.50	0.15	0.42	0.09
30	BH	*18.34*	*0.46*	*0.20*	*0.352*	*0.10*
	BY	*10.00*	0.36	0.14	0.25	0.07
	SimBa	10.03	0.36	0.14	0.25	0.07
75	BH	*86.93*	*0.59*	*0.29*	*0.43*	*0.15*
	BY	*27.43*	0.37	*0.15*	0.23	*0.07*
	SimBa	31.81	0.39	0.16	0.25	0.07

From these results, we see the positive relationship between the FDR and sensitivity across the competing methods. Because of this relationship, our choice of method for controlling the FDR presents a trade-off between higher sensitivity and a lower FDR. When using the more expansive definition of a true positive (a course with at least 33% of its students spiked), the FDR expectedly improves but the sensitivity worsens. The BH method always has a better sensitivity and a worse FDR than the other two methods because it flags more courses due to its anti-conservative nature. As the number of spiked courses increases (increasing the positivity rates in nearly all other courses), it is increasingly difficult to control the FDR. A lower spike factor (SF) in Table 5 makes it more difficult to identify problematic courses correctly and to differentiate performance between the three methods.

When there is only a small number of courses where increased transmission is occurring (i.e. 10 spiked courses) or the effect of the spiking is less dramatic (i.e., the spike factor is set to 2 as in Table 5), the BY method and the SimBa method perform essentially the same. However, as the number of spiked courses increases, our new simulation-based method (SimBa) differentiates itself from the Benjamini-Yekutieli (BY) method, behaving more like the less-conservative Benjamini-Hochberg (BH) method. From the simulation results, we conclude that for lower levels of transmission (in terms of both the number of hotspots and the severity of transmission increases within a hotspot), our simulation-based method provides the desired balance between the very conservative BY method and the BH method, which is rendered anti-conservative by the the violation of the standard assumptions associated with the method. We use the SimBa method to control the FDR in the (anonymized) real data analysis in the subsequent section.

## 5 Hotspot detection using Fall 2020 data

### 5.1 Risk comparisons

One way to evaluate the risk associated with membership in a university class is to compare it to the risk associated with a housing area, where the creation of social networks is facilitated and the COVID-19 virus spreads. If membership in a particular class had the same level of risk as membership in a particular housing area group, this would be of great concern to university administrators. While it would be infeasible or even unethical for administrators to close dorms or other housing areas as a response to an outbreak in a housing area, substantial evidence of outbreaks in classrooms could feasibly prompt a shift in learning mode (such as a move to remote learning). Knowledge that in-person attendance generally adds significantly to a student’s risk of infection can be justification for such academic disruption, while a class-specific elevation in risk could warrant class-specific intervention. We therefore propose to contextualize in-class risk by comparing it to other known vehicles for disease transmission, such as the risk associated with one’s housing group. We acknowledge that even if we assumed all environments are equally conducive to the spread of COVID-19, one would expect a randomly chosen housing area to have more frequent outbreaks than a randomly chosen class, due to the general tendency to spend a larger amount of time in one’s housing group, relative to the amount of time spent with one’s classmates. Notwithstanding, our analysis is a useful metric for characterizing relative risk as a criterion for intervention by university administrators.

To compare hotspot rates between sections of university courses and housing groups, we use a “risk ratio,” which compares the probabilities or likelihoods of disease among two populations. For our purposes, we define the risk ratio

$$\begin{array}{c} {RR}_{k}=\frac{L_{H,k}}{L_{S,k}}, \end{array}$$ (8)

where *k* is the time window of interest and *L*_*H*,*k*_ and *L*_*S*,*k*_ are the likelihoods or probability of being flagged as a COVID-19 hotspot (with time window of interest *k*) for housing groups and university course sections, respectively. We define *L*_*H*,*k*_ and *L*_*S*,*k*_ below. A ratio (*RR*_*k*_) value close to 1 indicates little difference between rates of the two groups while values above or below 1 indicate an increased or reduced risk.

To define *L*_*H*,*k*_ or *L*_*S*,*k*_, we let *x*_*G*,*k*,*t*_ be the number of hotspots flagged in grouping type *G* (where *G* is either *S* for “sections” or *H* for “housing areas” during week *t* using a time window of length *k*. We define the likelihood

$$\begin{array}{c} L_{G,k}=\frac{1}{N_{G}T}\sum_{t=1}^{T}x_{G,k,t} \end{array}$$ (9)

where *T* is the total number of weeks in a semester and *N*_*G*_ is the number of groups for that grouping type. For sections, *N*_*S*_ = 5708, and *N*_*H*_ = 12 groups for housing areas. The likelihood *L*_*G*,*t*_ creates an estimate for the average probability a group is flagged as a hotspot in any week of the semester. For example, if there were a total of 48 times that a sections was flagged as a hotpots during a 15-week semester, then the likelihood of a section begin flagged would be $L_{S,15}=\frac{48}{15(5708)}=0.0005606=0.056\%$. For housing areas, if there were a total of 18 times a housing group was flagged as a hotspot during a 15-week semester, the likelihood of a housing area being flagged as a hotspot would be $L_{H,3}=\frac{18}{15(12)}=0.1=10\%$.

In computing *L*_*H*,*k*_, we model hostpots among housing groups in the same way we have done for sections. The groups in our null hypothesis are now housing areas (as defined in the third bullet of Section 2.1), and in our XGBoost model, we use only variables that do not have any association with housing groups. In turn, we now create variables for evaluating *PPP*_*i*_ that pertain to a students schedule such as the average positivity rate of sections, the maximum positivity of sections in a students schedule, total number of positives in students schedule, and others.

As part of the null hypothesis for detecting housing group hotspots, we are careful in computing features where a students positivity status may be used in its formation. That is, we avoid unintentionally embedding a students positivity status in a feature used in an XGBoost that predicts a student’s positivity status.

Once *L*_*H*,*k*_ and *L*_*S*,*k*_ have been estimated, we compare the relative risk between the two groups with *RR*_*k*_ and a 95% confidence interval (CI). If the interval covers 1, we have reason to believe that attending a particular class and living in a particular housing area have similar COVID-19 risk levels.

It should be noted that typical risk ratios compare the relative risk among two separate, independent groups. In our setting, the two sets of “groups” are housing areas and sections, but they are formed from the same population of students. Despite our nonstandard use of these relative risks for various groupings of students, we argue that they are useful and appropriate for characterizing the risks associated with these partitionings of a population. Calculations using a student’s neighborhood are used when predicting *PPP*_*i*_ for modeling sections, but this is done at the individual level without knowledge of the student’s larger identification in a housing group.

### 5.2 Results: Model fit

**Fig 5:**
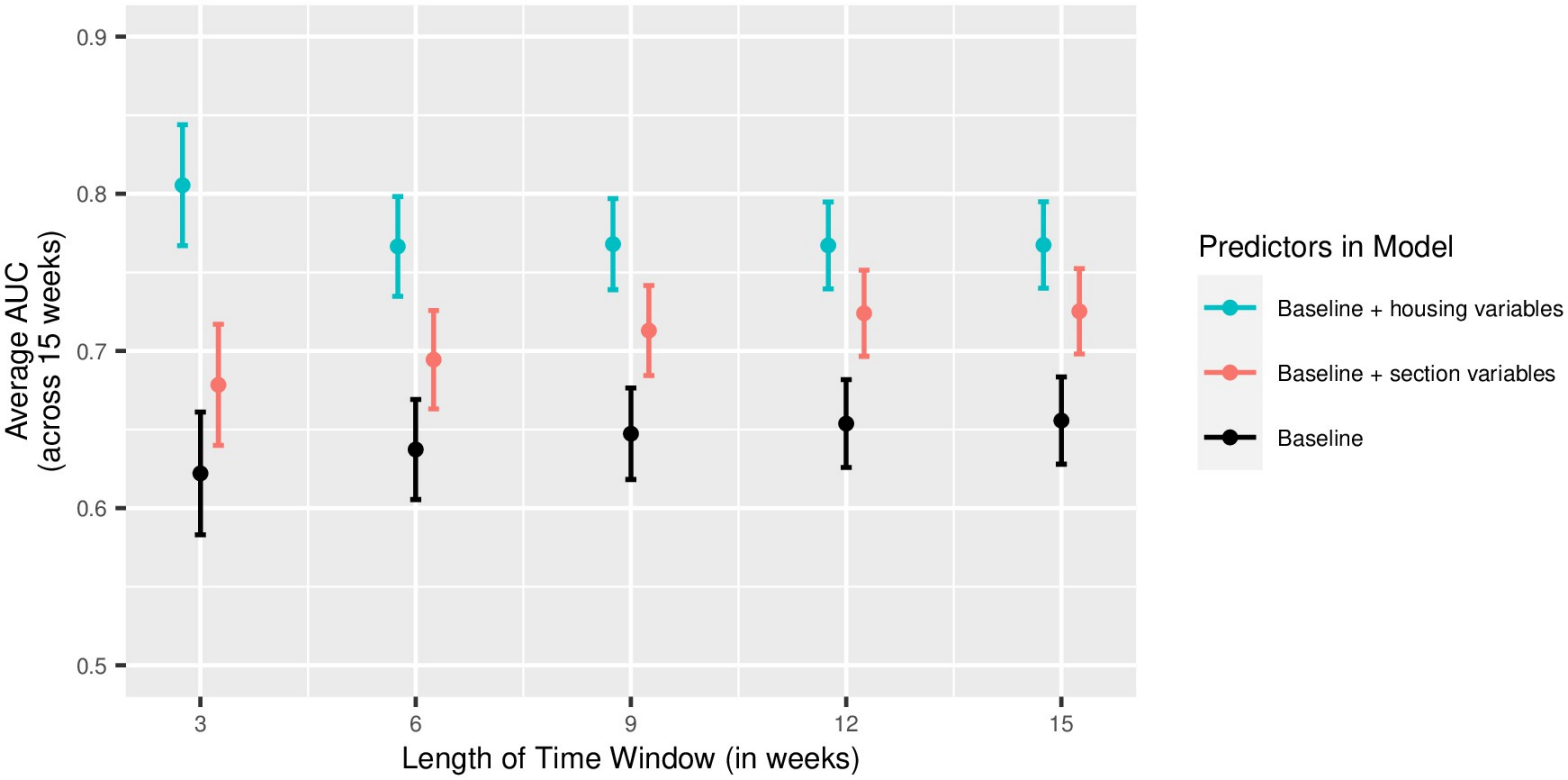
AUC performance by group to be analyzed. Average AUCs for an XGBoost model trained across 15 weeks of Fall 2020 for various time window lengths. The color of the dot (and 95% confidence interval) correspond to the group the XGBoost probabilities are used to simulate: blue for sections and red for housing groups. The estimates and intervals shown in black show the performance of an XGBoost model using only basic demographic information from the university.

A separate XGBoost model was trained for housing groups and sections each week. The average AUC for an XGBoost model across a 15 week semester with time windows *t* = 3, 6, 9, 12, and 15 weeks are shown with dots in Fig 5. The bars extending from each dot show a bootstrapped 95% CI around the AUC estimate, and the black, dashed line shows the average AUC when only the initial (baseline) information from the university was used each week.

In our case, AUC performance is an indicator of risk predictiveness, given the variables used for prediction. That is, if a model was highly predictive of COVID-19 status, the variables used would be a powerful source for quantifying COVID-19 risk. Under the null hypothesis (for modeling either sections or housing groups), variables associated with that group in any way are not used. (For example, we do not use COVID-19 rates from a student’s class schedule in a model when evaluating the potential for hotspots in sections). In this setting, the XGBoost models were more predictive of COVID-19 risk when a student’s local housing information was included in the model (as shown by the blue 95% CIs in Fig 5), regardless of time window. In other words, using housing information to model a student’s risk was much more successful than using section information to model a student’s risk. XGBoost models using neither section nor housing group information performed most poorly.

### 5.3 Results: Hotspot detection

#### 5.3.1 Quantifying hotspots

**Fig 6:**
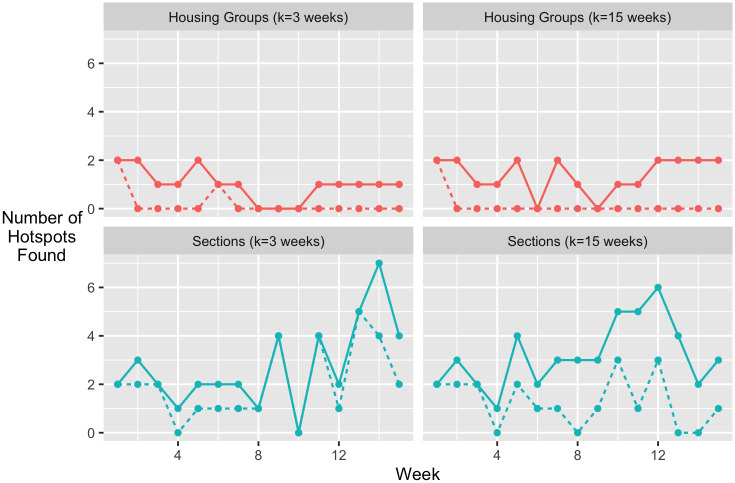
Hotspot counts. Counts of hotspots for housing groups (top plots) and sections (bottom plots) for each week of the Fall 2020 semester. Two different lengths of time window are compared: *t* = 3 (left plots) and *t* = 15 (right plots). Solid and dashed lines denote the total and unique count, respectively, for a given week.

Having trained the final models, we performed the Monte Carlo simulations, *p*-value correction, and counted the groups flagged as hotspots for both sections and housing groups. The number of groups that what were flagged as hotspots each week for sections and housing groups using a time window of 3 and 15 weeks are shown in Fig 6 across the 15 weeks. Solid lines show the unadjusted total count of groups flagged as hotspots, meaning that a group flagged as a hotspot in multiple weeks is counted multiple times. Dashed lines show the weekly count of new hotspots, including only unique groups that have not been flagged previously in the semester. Note that the most likely time to find a housing area hotspot was in the initial weeks, while the most common time to find a hotspot in a section was the closing weeks of the semester.

In the top row of Fig 6, we note that a few housing groups are flagged early in the semester and remain problematic for several weeks. In the bottom left plot of Fig 6, the unique and total counts of flagged sections follow each other closely. This means newer sections are being flagged each week, rather than the same sections remaining problematic throughout the semester. When the time window is extended to 15 weeks, we see something similar to the trend that occurred within housing groups; that is, a smaller number of groups being flagged multiple times throughout the semester.

Table 6 shows the counts and likelihoods of hotspots for sections and housing groups for different time windows. The unique and total count are as explained above, but summed across the whole 15-week semester. For example, when *k* = 6, there were 24 sections flagged a total of 51 times as hotspots across Fall 2020. Note that shorter lengths for the time window of interest have little effect on the total sections flagged, but yield a higher number of unique sections flagged. This is unsurprising, as spikes in COVID positive students elevating a section up to hotspot status will be forgotten with a narrower moving window. The likelihoods of a hotspot for sections and housing groups are also shown in Table 6, as is the relative risk of housing groups relative to sections (with a 95% CI).

**Table 6:** Counts and likelihoods of hotspots flagged for sections and housing groups using various time windows (*k*). The unique count is the unique number of groups flagged, and the total count is the total number of groups flagged (may be flagged more than once) across a 15-week semester. Using time window *k*, *L*_*S*,*k*_ and *L*_*H*,*k*_ are the likelihoods of a hotspot group for sections and housing, respectively. The relative risk of the two groups *RR*_*k*_ (from Eq 8) is also shown with a 95% confidence interval (CI).

	Sections (*N*_*S*_ = 5708)			Housing Groups (*N*_*H*_ = 12)			
Time Window (*k*)	Unique Count	Total Count	*L*_*S*,*k*_	Unique Count	Total Count	*L*_*H*,*k*_	*RR*_*k*_ (95% CI)
3	30	41	0.0479%	4	18	8.33%	174 (98.1, 309)
6	24	51	0.0596%	4	27	9.44%	159 (93.4, 269)
9	22	42	0.0491%	3	23	9.44%	193 (112, 332)
12	19	47	0.0549%	3	21	10.6%	192 (115, 321)
15	19	48	0.0561%	3	19	11.7%	208 (127, 340)

#### 5.3.2 Risk comparisons

**Fig 7:**
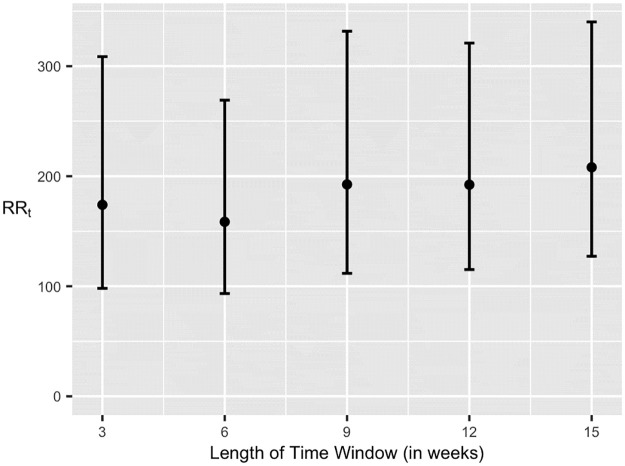
Risk ratio for sections and housing groups. Estimated “Risk Ratio” using *L*_*H*_ and *L*_*S*_ for time intervals *k* = 3, 6, …, 15 weeks. Vertical bars form 95% confidence intervals for each estimate. Values covering to 1 indicate little difference between likelihoods for housings and sections to be flagged as hotspots.

Finally, we compare the relative risk of housing groups to sections. We computed the risk ratio *RR*_*k*_ for *k* = 3, 6, …, 15 weeks. This is shown in Fig 7 (exact estimates can be found in the final column of Table 6). Estimates are shown with a black dot with a 95% CI extending vertically in black. Each 95% CI was formed on the log(*RR*_*k*_) scale (where log is base 10) then back transformed to the original scale.

These results show that housing areas were roughly 100 to 300 times more likely to be flagged as a hotspot than a classroom in Fall 2020. In other words, relationships within housing groups are much more likely to increase COVID-19 risk than relationships within classrooms during that semester. Such calculations are highly valuable to university administrators deciding whether class should be moved to remote learning or continue in person. It shows that pandemic safety protocol practiced in classrooms might be working well when compared to behaviors of the same population outside of campus. These analyses lend some credence to the supposition that, in general, associations with other students in a class is less concerning than associations with those in home or social environments.

## 6 Conclusion

Our proposed approach provides a framework that enhances the ability of college and university administrators to properly flag a group as high risk and intervene accordingly. Beginning with a model for individual positivity probabilities, our approach allows for any group to be analyzed by considering its individual group members in a Monte Carlo setting. This framework can be especially effective during the volatile stages of a pandemic: when educators want to remain with in-person learning and its advantages, but want to intervene with or notify high-risk groups in some way (e.g., to move a section to remote instruction for two weeks). Our approach allowed administration to intervene in a targeted fashion (e.g., by working with course instructors or housing complex managers), as opposed to imposing a universal and academically disruptive intervention. Although it is difficult to quantify the value added by the use of hotspot tracking, we note that the University studied was able to keep classes open throughout the 2020-2021 and 2021-2022 academic years. Along with common COVID-19 safety practices (physical distancing and mask-wearing), these methods allowed university officials to monitor specific hotspots until positivity rates diminished.

We recognize several limitations of this research. Even though a percentage of all students were randomly selected for testing each week during Fall 2020, most of the data were from self-reports or COVID testing facilities. Consequently, the data exhibit substantial bias due to unreported or unknown cases. Having more accurate information about the actual number of cases on campus would certainly improve our results. Additionally, any predictive model may perform well here, not just XGBoost. Future research could be aimed at trying different statistical, machine learning, or deep learning models to increase predictive capabilities. Improvements in classifying the COVID-19 status of a student and evaluating personal risk levels will increase the sensitivity of our analysis.

Hotspots were frequently identified within housing areas where social interactions are known to be frequent and in intimate settings. However, hotspots were relatively rare within sections of university courses; compared to any one section, any one housing area was more than 100 to 300 times more likely to become a hotspot. While the number of hours a student spends at home will dramatically exceed the number of hours spent in particular class, characterizing this comparison can be helpful to university administration. For the majority of students living in high-density housing, learning in carefully-controlled classrooms may have been safer than learning at home where social mixing is pervasive and physical distancing and mask-wearing were less common. Methods such as these that allow the comparison of relative risks across different groups have proven useful for university leaders in making decisions about learning modes (i.e., in person versus online).

As noted by one of the anonymous reviewers of this article, the spatial locations of classes may be a helpful source of information about disease spread. For example, a poorly ventilated building may contribute to several sections having higher than expected COVID rates. One complication for using classroom locations is that buildings are so closely confounded with the disciplines (majors) that attend class there, confounding the effects of building and college major. As such, we included college-major positivity rates as features in our machine learning model and then considered the location of flagged courses post-hoc. We did not observe any clustering of classroom hotspots within particular buildings and therefore maintained focus on surveillance of possible hotspots within class sections, majors, and housing areas.

We note here that although our motivation and data are associated with COVID-19 in a university setting—and with students particularly, the proposed methods described here can be applied to an entire university community including faculty, staff, administrators, and other non-students or to any multi-faceted network of persons. For example, a large corporate office, warehouse, manufacturing facility, or branch of government might be interested in identifying potential hotspots in its network—comparing different shifts, office locations or floors, or interdisciplinary working groups. A county health department may wish to identify hotspots within neighborhoods, public schools, or age groups. These methods also apply to the tracking of hotspots for any infectious disease or even for information that is socially-transmitted through parts of a network.
